# Dental Sealant Composition-Retention Assessment in Young Permanent Molars

**DOI:** 10.3390/ma14071646

**Published:** 2021-03-27

**Authors:** Alexandrina Muntean, Codruta Sarosi, Sorina Sava, Marioara Moldovan, Andrei Ilie Condurache, Ada Gabriela Delean

**Affiliations:** 1 Department of Paediatric Dentistry, Iuliu Hatieganu University of Medicine and Pharmacy, 31 A. Iancu Street, 400083 Cluj-Napoca, Romania; ortoanda@gmail.com; 2 Institute of Chemistry Raluca Ripan, Department of Polymer Composites, Babes-Bolyai University, 30 Fantanele Str., 400294 Cluj-Napoca, Romania; mmarioara2004@yahoo.com; 3 Department of Prosthodontics and Dental Materials, Iuliu Hatieganu University of Medicine and Pharmacy, 15 V. Babes Street, 400012 Cluj-Napoca, Romania; 4 Resident Physician—Laboratory Medicine, Cluj County Clinical Hospital, 3-5 Clinicilor Street, 3-5, 400000 Cluj-Napoca, Romania; conduandrei@yahoo.com; 5 Department of Conservative Dentistry, Iuliu Hatieganu University of Medicine and Pharmacy, 33 Motilor Street, 400001 Cluj-Napoca, Romania; adadelean@yahoo.com

**Keywords:** dental sealant, retention, molar morphology and topography

## Abstract

Tooth decay in children and adolescents remains a public health problem, despite prophylaxis and preventive measures being largely available. The aim of our study was to evaluate the clinical behavior of four dental sealants, related to first permanent molar topography and patient age (when sealant was applied for the first time). We assessed, by means of visual inspection and palpation with a dental probe, a group of 200 children, enrolled corresponding to school age-grade (mean age of 7 years at baseline) and randomly divided according to the material used as dental sealant (Admira seal©, Embrace Wet Bond©, Fotoseal©, GC Fuji Triaje©) in 4 groups (*n* = 50). Sealant clinical evaluation was made at 6-, 12-, 18-month intervals for dental material retention assessment. At 6 months, the sealant detached the most from 3.6 molars, and the material used was Fotoseal© (27.6%). At 12 months, Fotoseal© (48.3%) and GC Fuji Triaje© (41.4%) from 3.6 molars express detachment. At 18 months, 4.6. molars sealed with Admira Seal© (25.7%) and Embrace Wet Bond© (28.6%) lost the sealant. We noticed less detachment in maxillary molars and if sealant was applied around 7 years of age. In conclusion, sealant application on first permanent molars must be encouraged and practitioners can choose between various materials available.

## 1. Introduction

Dental caries is a biofilm-triggered oral disease with an international pandemic distribution, affecting all age children and adolescents [1]. The World Health Organization reports that dental caries is the most common oral condition included in the Global Burden of Disease Study [2]. Carious lesions of permanent teeth represent the most prevalent oral condition, while carious lesions on deciduous teeth ranks 12th [2]. 

Tooth decay in children and adolescents remains a public health problem, despite prophylaxis programs and preventive measures being largely available (dental sealants, personal and professional hygiene and healthy dietary habits) principally as pits and fissures on children’s teeth; this presents a topography with deep and narrow features, making the mechanical removal of dental plaque, by brushing, very challenging [3]. Sealing pits and fissures were introduced in the 1960s to protect from caries and include the placement of a fluid material onto the occlusal surface of posterior teeth and forming a layer that is bonded micromechanically and acts mainly as a barrier against acids and the subsequent tooth mineral loss [4,5]. Sealing materials are considered to be the best caries prophylaxis agents in pit and fissures because negative morphological details favor bacteria and food remnants retention, rendering correct oral hygiene difficult [4,6]. Reduced salivary access into the fissures diminishes the fluoride effectiveness at this spot. Probably, the most caries-susceptible phase of first permanent molar is the long eruption phase, as the enamel is immature during this period [7,8]. Sealant application is highly effective in occlusal caries prevention; 71% of occlusal decay is preventable after one-off sealant application [9]. Progress in dental materials and the development of pit and fissure sealants, have shifted the treatment philosophy from “drill and fill” to “seal and heal” [8]. Dental sealants are recommended on temporary and permanent teeth, even on those who have non-cavitary lesions, to stop their progress [3,4,8]. Adequate pit and fissure sealant use has been demonstrated to be effective in occlusal caries prevention; incorrect application of the sealant could result in microleakage, sealant loss and decay initiation. In order to prevent these complications, regular follow-up is required to ensure long-term success of the method [9].

Romanian adolescents between 10 and 19 years have a high caries prevalence (95.5%) and a DMFT index of 3.13 [10]. 

Regarding the oral health status of 6- and 12-year-old children in the Transylvania and Central Region of Romania, ICDAS caries codes more prevalent in 6-year-old children were “0A” (first visual change in enamel—34.33%), followed by “06” (tooth with extensive distinct cavity—29%) [11].

Under these circumstances, we consider that prophylactic interventions, such as dental sealants, must be considered and used more frequently.

Dental sealants belong to several categories of dental materials, each with advantages and disadvantages. The widest class of materials used as sealants, dental composites (flow composites, nanocomposites or ormocers), have as their main advantage a good enamel retention rate and long-lasting mechanical protection [12,13,14,15]. Etching the enamel surface prior to sealant application ensures adequate retention, but can be challenging in the distal area of the dental arch. To enhance the prophylactic effect, various physical and chemical properties of resin-based sealants were proposed, in order to allow the application on newly erupted teeth, which cannot be perfectly isolated [12,14].

Glass ionomer cements reveal qualities that allow their application in situations when moisture control cannot be achieved properly. These materials also report chemical bonding to enamel and act as dynamic element for fluoride balance in the oral cavity. No dental material exerts ideal properties and for this reason sealants must be selected in relation to every clinical situation [16,17,18]. Dental sealants’ effectiveness was widely investigated in terms of retention, mechanical properties, marginal microleakage, infiltration and the emergence of caries [19,20].

The aim of our study was to assess dental sealants retention depending on molar position on dental arch and material characteristics. The null hypothesis was that patient age, when dental sealant was first applied, did not affect retention during 18 months.

We aim to encourage dental sealants use, with no special dental office condition and reveal that adequate results in terms of retention and occlusal decay control can be achieved.

## 2. Materials and Methods

The study was conducted over a period of 18 months for with a group of 200 children attending a primary school in Cluj-Napoca city, Romania. Children were examined in the school’s dental office, as part of annual health assessment. The children were enrolled, prior to parent’s informed consent (in respect to the application of tooth decay preventive methods).

The children were selected based on their school age, first elementary class, which corresponds to an average age of 7 years (Table 1).

This age was chosen because once the children commence school (and it has a dental office), the periodic control is performed at the beginning of each semester, facilitating the patient monitoring. In the absence of prevention programs, children’s access to specific methods of dental caries prophylaxis is limited in our country. A specific prophylactic method was proposed to all children enrolled in first elementary class (*n* = 218) but parents’ consent was only obtained for *n* = 200 children.

Inclusion criteria:

- Normal, healthy children;
- Availability for the duration of the study;
- Satisfactory dental care performed at home;
- The willingness of the patient to accept the treatment.

Exclusion criteria:

- Enamel dysplasia, MIH;
- A child with compromised health;
- Long-term medication affecting the salivary flow;
- Adverse reaction reported to any dental material;
- Uncooperative child.

The study was conducted with the agreement of the Ethics Commission of the University of Medicine and Pharmacy, Iuliu Hatieganu, Cluj Napoca, no. 221/17.05.2017.

The children selected based upon above-mentioned criteria were then randomly divided into four equal groups (*n* = 50) depending on sealant used. First permanent molars were assessed by a single examiner in school’s dental office, using visual and tactile examination methods. First permanent molars with an occlusal surface accessible for isolation, free of cavities, were sealed with the specific sealant (Table 2).

### 2.1. Sealing Procedures.

**Figure 1:**
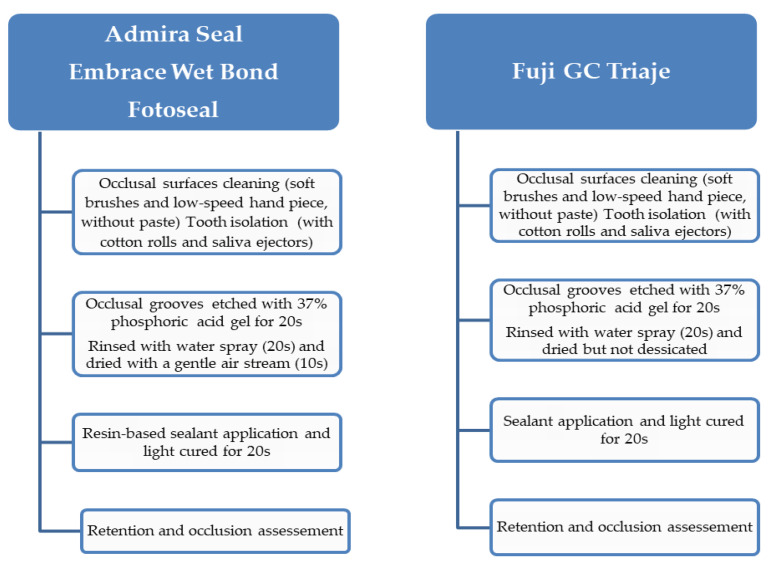
The schematic representation of sealing procedure.

Occlusal surfaces were cleaned by employing soft brushes and a low-speed hand piece, without paste. Tooth isolation was achieved with cotton rolls and saliva ejectors, held by an assistant. Sealants were placed in respect to the manufacturer’s recommendations. The schematic representation of sealing procedure is presented in Figure 1.

- Group I–III: the occlusal mesio-distal grooves were etched with 37% phosphoric acid gel for 20 s (Vococid©, Voco Corporation, Cuxhaven, Germany), rinsed with water spray for 20 s and dried with a gentle air stream for 10 s. One layer of resin-based sealant was applied using a special applicator with a light brushing motion, and then light cured for 20 s using LED Elipar Deep Cure-L© (3M, St. Paul, MN, USA)
- Group IV: the occlusal mesio-distal grooves were conditioned with GC Cavity Conditioner©, GC America INC, Alsip, IL, USA (20% polyacrylic acid and 3% aluminum chloride hexahydrate) for 10s, then washed for 20 s and dried but not desiccated with a gentle air stream for 3 s, in order to obtain a moist surface. The resin-modified glass ionomer sealant (RMGI) (GC Fuji Triage^®^, GC America INC, Alsip, IL, USA) was prepared according to the manufacturer’s recommendations and one layer was applied with an explorer, and then light cured for 20s using LED Elipar DeepCure-L© (3M, St. Paul, MN, USA).

The light-curing unit was operated in the standard mode at a light intensity of 1470 mW/cm^2^ (−10%/+20%). The light curing unit output was measured as specified by the producer.

The immediate retention was verified with an explorer and the occlusion was tested with articulation paper. Children were instructed not to eat or drink for 30 min.

All fissure sealants were placed by one pediatric dentist with an assistant. Post-sealing valuations were performed by a single examiner, in the same dental office, in similar conditions.

At each recall visit (at 6-month intervals), all sealed occlusal surfaces were examined with an explorer for dental material retention assessment. The examined surfaces, according to dental sealant retention, were considered as follows:

- Sealant completely preserved;
- Incomplete or complete missing material, without caries;
- Incomplete or complete missing material, with caries.

In teeth with partial or total loss, fissure sealants were re-applied as needed, if no sign of carious lesion was detected. Re-sealed surfaces were included in further evaluation.

We used 6-month interval assessment on the basis that routine recall examinations for children must be twice a year. The follow-up period included 18 months of preventive measures, with their effects expressed in relation to time and school policy, allowing parents to move the children to another school.

### 2.2. Data Analysis

Data were analyzed using IBM SPSS Statistics 25 (SPSS Inc, Chicago, IL, SUA). Quantitative variables were tested for normal distribution using the Shapiro–Wilk Test and were written as averages with standard deviations. Qualitative variables were tested for differences between groups using Fisher’s Exact tests and were written as counts with percentages. Quantitative variables with non-parametric distribution were tested between groups using Mann–Whitney U and Shapiro–Wilk Test. Z-tests with Bonferroni correction were made to further analyze the results. The results were considered significant for *p* < 0.05.

All the data from the study was analyzed using IBM SPSS Statistics 25 and illustrated using Microsoft Office Excel/Word 2013, Microsoft Corporation, Redmond, WA, USA. Quantitative variables were tested for normal distribution using the Shapiro–Wilk Test and were written as averages with standard deviations. Qualitative variables were tested for differences between groups using Fisher’s Exact tests and were written as counts with percentages.

Quantitative variables with a non-parametric distribution were tested between groups using Mann–Whitney U. Z-tests with Bonferroni correction were made to further analyze the results obtained in contingency tables.

## 3. Results

**Figure 2:**
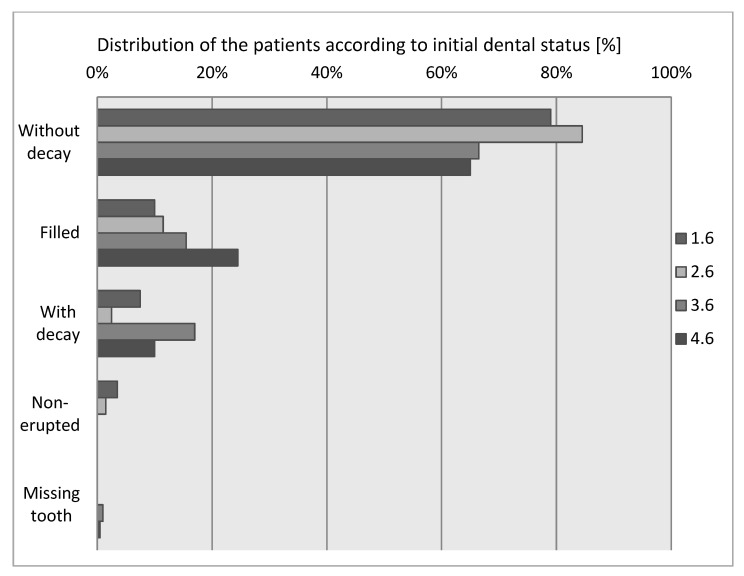
First permanent molar status-initial assessment.

At the beginning of the study, a number of first permanent molars showed untreated active caries, fillings or had already been extracted. The numerical distribution of the first permanent molars according to the topography on the arch and the dental status was as follows (Figure 2).

We noticed that most of the examined patients had their first permanent molars present, without decay when the study was initiated. Within the teeth, it can be seen that 4.6 was the most frequently filled tooth, followed by 3.6 while 1.6 and 2.6 were the most rarely filled teeth. The 3.6 molar was the most frequently observed as having decay at the initial examination. Overall, 3.5% (1.6) and 1.5% (2.6) of first permanent molars were not erupted at the initial examination; 1% (3.6) and 0.5% (4.6) of teeth were missing due to extraction.

For all school children, all caries-free permanent molars were sealed with the selected sealant (according to inclusion group).

### 3.1. Sealant Retention in Relation to Dental Material Characteristics.

The condition of the sealed surfaces was examined immediately after procedure, by gentle inspection and palpation with the probe, and at 6-, 12- and 18-month intervals. Sealant retention acts as a key factor for occlusal surfaces prophylaxis. Immediate retention for sealed surfaces in our study was 100%.

**Figure 3:**
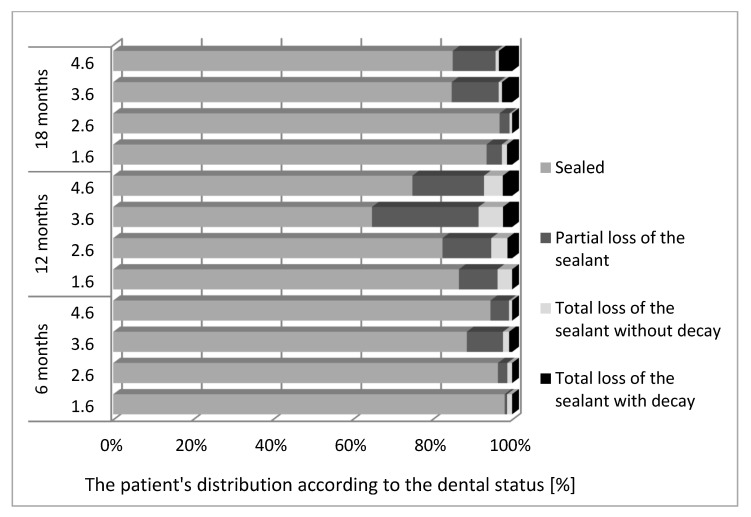
Dental sealant retention assessment at 6, 12 and 18 months.

We notice, in our study group, that an important percentage of sealed surfaces remain intact at regular follow-up, for both upper and lower molars (Figure 3).

At 6 months, partial loss of the sealant was detected, especially for 3.6 and 4.6. At 12 months, partial loss of the sealant, without decay, was noticed to be more important for both maxillary and mandibular molars. At 18 months, the values decrease, but in mandibular arch partial and total loss of the sealant appear to be more important.

Sealants evaluated in our study react differently in accordance with first permanent molar position on dental arch. Sealant lost from occlusal surface, as differences between materials, was analyzed with Fisher’s test and Z-tests.

**Figure 4:**
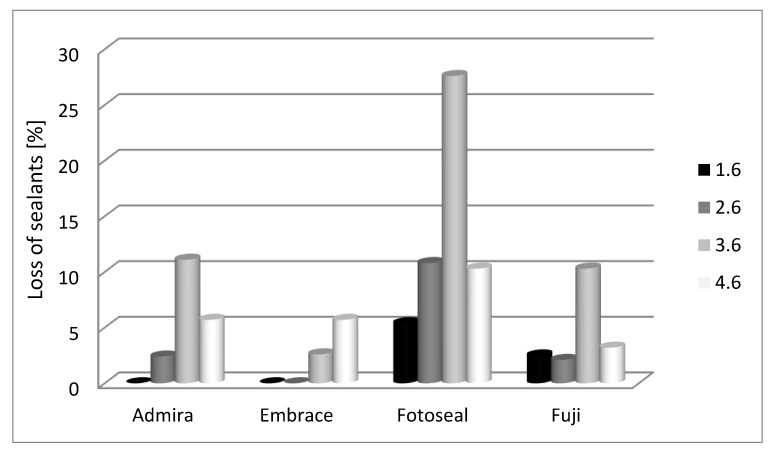
Dental sealant lost in accordance with first permanent molar topography—6 months assessment.

At 6-month evaluation, mandibular molar appeared to be more affected when compared with maxillary molars (Figure 4).

Disparities between the percentages of lost sealant for 1.6 molar are not significant between the studied materials (*p* = 0.219); we discern that only Fotoseal© (5.4%) and Fuji GC Triaje© (2.5%) materials express detachment. Conversely, there are significant differences for 2.6 molar between the studied materials for Fotoseal© (10.8%) (*p* = 0.037).

For 3.6 there are significant differences between the percentages of lost sealant only for Fotoseal© (27.6%) (*p* = 0.016). There are not any significant differences for 4.6 molar between the studied materials (*p* = 0.705) although it is worth mentioning that Fotoseal© (10.3%) was the most affected material.

**Figure 5:**
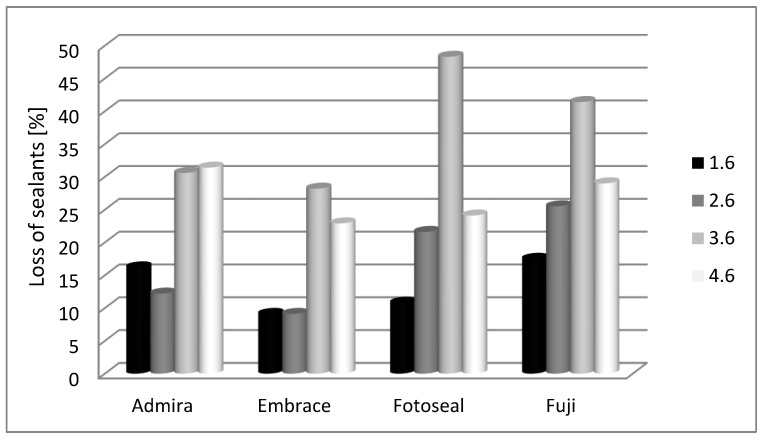
Dental sealant lost in accordance with first permanent molar topography—12 months assessment.

At 12-month evaluation, values for sealant loss were more important for mandibular molars when compared with maxillary molars (Figure 5).

For 1.6, there are not any significant differences between the studied materials (*p* = 0.631); we notice that Admira Seal© (16.2%) and Fuji GC Triaje© (17.5%) were the most affected materials. There are not any significant differences for 2.6 in terms of sealant loss (*p* = 0.136); Fotoseal© (21.6%) and Fuji GC Triaje© (25.5%) materials were the most affected.

For 3.6 molars, there are not significant differences between the studied materials (*p* = 0.307), we detect that Fotoseal© (48.3%) and Fuji GC Triaje© (41.4%) were the most concerned materials. For 4.6 molars, the differences were non-significant between the studied materials (*p* = 0.834); we discern that Admira Seal© (31.4%) and Fuji GC Triaje© (29%) were the most affected.

**Figure 6:**
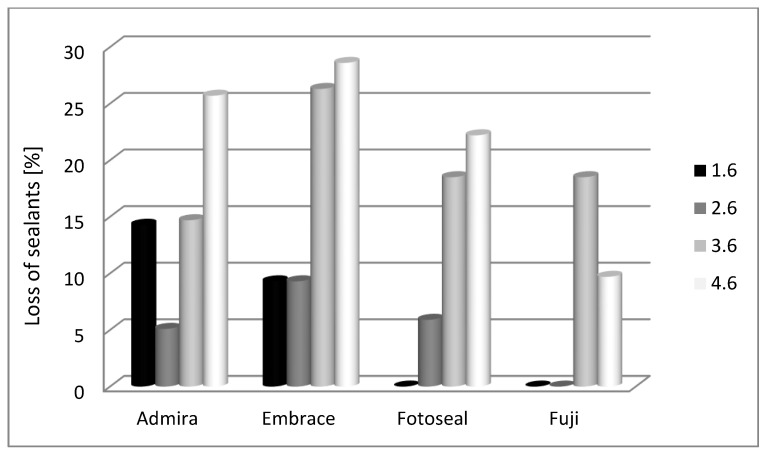
Dental sealant lost in accordance with first permanent molar topography—18 months assessment.

At 18 months, mandibular molars express deficient retention when compared with maxillary molars (Figure 6).

Significant differences for 1.6 were obtained at 18 months for Admira Seal© (14.3%) (*p* = 0.022) when compared with Fotoseal© (0%) or Fuji GC Triaje© (0%). Other differences between Admira Seal© (14.3%) and Embrace Wet Bond© (9.3%) were not statistically significant.

The comparison between sealant materials for 2.6 molar showed that there are not any significant differences (*p* = 0.267). We notice that Embrace Wet Bond© (9.3%) and Fotoseal© (5.9%) were the most affected.

For 3.6 there are not any significant differences between the studied materials (*p* = 0.675); Embrace Wet Bond© (26.3%) was the most affected sealant.

For 4.6 molar at 18 months there are not any significant differences between the studied materials (*p* = 0.276). Most occlusal surfaces sealed with Embrace Wet Bond© (28.6%) and Admira Seal© (25.7%) lost the sealant.

Surfaces with sealant, partially or completely detached, if there were no signs of decay, were sealed again. If caries diagnoses were confirmed, filling was made.

In our study group, total loss of the sealant did not expose the occlusal enamel to caries initiation, an element that is in line with the literature data. During the follow-up period, decay incidence was very low; we assume that preventive methods, oral hygiene and regular dental check-up are responsible for these results.

### 3.2. Sealant Retention, Dependent on the Topography of the Tooth and Patient Age (When the Tooth Was Sealed for the First Time).

Individual factors (patient age, tooth morphology and position) can play an important role in long term sealant retention.

For 1.6, the distribution of the patients’ ages was detected as non-parametric (*p* < 0.05) according to the Shapiro–Wilk test (Table 3).

According to the Mann–Whitney U tests, age was not statistically significant when comparing patients who lost the sealant to those who did not for 1.6 molar.

According to the Mann–Whitney U tests, the differences between ages, taking into account the moment when the sealant was applied for the first time, did not express statistically significant differences (*p* > 0.05) in sealant retention, during the follow-up period, for 1.6 molar.

For 2.6, patients age distribution was detected as non-parametric (*p* < 0.05) according to the Shapiro–Wilk test. (Table 4)

According to the Mann–Whitney U tests, the differences between ages, considering the moment when the sealant was applied for the first time, did not express statistically significant differences (*p* > 0.05) in sealant retention at 12- and 18-month assessment, for 2.6 molar.

At 6 months, patients who lost their sealant partially or totally were significantly older (median age = 8 years) than patients who had their sealant intact (median age = 7 years) in our study group, and we can assume that for 2.6 it would be better to apply the sealant at a younger age, specifically around 7 years.

For 3.6, the distribution of the patients age was detected as non-parametric (*p* < 0.05) according to the Shapiro–Wilk test (Table 5).

According to the Mann–Whitney U tests, the differences between ages, considering the moment when the sealant was applied for the first time, did not express statistically significant differences (*p* > 0.05) in sealant retention at 6- and 12-month assessment, for 3.6 molar.

At 18 months, patients who lost their sealant partially or totally were significantly younger than 7 years (median age = 7 years, interquartile range = 6–7 years) than patients who had their sealant intact (median age = 7 years). We can assume that for 3.6 molar, similar to the 2.6 molar, dental sealant applied around 7 years is associated with a higher rate of retention.

For 4.6, the distribution of the patients age was detected as non-parametric (*p* < 0.05) according to the Shapiro–Wilk test (Table 6).

According to the Mann–Whitney U tests, the differences between ages, considering the moment when the sealant was applied for the first time, did not express statistically significant differences (*p* > 0.05) in sealant retention at 6- and 12-month assessment, for 4.6 molar.

At 18 months, patients who partially or totally lost their sealant were significantly older than 7 years (median age = 8 years, interquartile range = 7,8 years) compared to patients who had their sealant intact (median age = 7 years). We considered that for 4.6 molar, similar to the 2.6 and 3.6 molar, first placement of dental sealant at 7 years is associated with a higher retention rate.

## 4. Discussion

The concept of risk-based sealant application can form the basis of the rationale for this specific prophylactic method in addition with other considerations like tooth morphology, caries incidence, fluoride history, oral hygiene, patient collaboration and dental material physical and chemical properties [19,21]. Fissure sealants have been established as clinically and financially effective interventions for the prevention of caries development in pits and fissures [21,22]. The retention of the sealant is traditionally considered as a measure for caries prevention and, thus, is widely regarded as the single most important outcome measure for the evaluation of fissure sealant success [23,24].

The current results of this study indicate that the partial or complete sealant loss does not comply with dental decay initiation, in line with literature data that state that the caries-preventive effect of the sealant must be analyzed not only from perspective of the retention rate. [21,25].

Research demonstrated the caries-preventive effect of sealant in comparison to no intervention and in line with these, patients may not really worry about how long fissure sealants are retained, but rather how long carious lesions in their teeth remain prevented. In this regard, oral care providers have an ethical duty to patients to offer interventions that have been shown to be effective in achieving their primary, patient-centered clinical outcomes, like the prevention of occlusal carious lesions [14,21,23].

The development of dental caries is relatively high during the eruption of permanent teeth and, as soon as possible, protective dental materials should be applied on occlusal surfaces accessible for this procedure; there exists information regarding the age when fissure sealant might be applied but individual caries risk and morphological and psychological factors must be considered [9,17].

The stage of tooth eruption, the behavior of the child, the possibility of adequate isolation (rubber dam or cotton roll) are factors that should be taken into account because fissure sealant fails to succeed mainly due to lack of adequate isolation and enamel contamination by saliva or gingival fluid [17,18]. In our study group, maxillary molars perform better than mandibular ones, and also a patient’s right side (more accessible in terms of access and isolation) reveals differences when compared to the left side.

All thorough dental material chemical composition and physical properties, acts as factors that enhance sealant adhesion and durability over time. Dental sealants must reliably access the enamel, rendering the region beneath accessible to cleaning procedures and less prone to demineralization or caries attack.

The isolation performed by a cotton roll and saliva ejector proved to be accurate in our study. We noticed that the mandibular molars needed retreatment more often than maxillary molars. This may be due to newly erupting mandibular permanent molar, the distal tissue flap or operculum of which seemed to be present for a longer period thereby making isolation of the occlusal surface more difficult [18,20].

The adhesion of the resin to the enamel depends not only on the application of acid etch to the enamel but also on other factors like polishing of the dental surface prior to etching, etching time, the concentration of the acid and the type of acid used. Regarding etching time, many authors have already advocated a reduction in time since they observed no difference in the adhesion of sealants and they all recommended a standard time of 15 to 20 s; we have used a standard time of 20 s for each tooth before sealant placement [18,20,21].

Glass ionomers bond to both enamel and dentin by physicochemical mechanisms following polyacrylic acid conditioning. Inadequate isolation can compromise long-term retention even for glass ionomer cements or resin-based, moisture-tolerant sealants [15,16,21,26]. Filled resin-based sealants cannot penetrate deep and wide fissures because they have a relatively large hybrid monomer [25,26,27]. Adequate curing of the material is important for success. The curing light should be tested to make certain that intensity is optimal. Accidental contamination of saliva, particularly when treating newly erupted permanent molars in young children could partially explain the reason for poor retention rates [18,21]. Improper marginal sealing can cause the bacteria and its fluids to penetrate the sealant–tooth margin, increasing the incidence of recurrent caries beneath the sealant [22,23]. For the long-term success of pit and fissure sealants, retention and proper adhesion to enamel surface are mandatory [24,25]. Filled resin-based sealants cannot penetrate deep and wide fissures when compared with filled material. Additionally, inadequate isolation can compromise long-term retention even for glass ionomers (GI) or resin-based (RB) moisture-tolerant sealants. The glass ionomer sealant appeared to have four times higher chance of preventing caries development in re-exposed pits and fissures of occlusal surfaces in first molars than composite resin sealant material [28,29].

Partial or total dislocation of the sealing material does not cause an immediate increased caries susceptibility of the enamel surface because, in the occlusal surface morphological details, the remaining material provides anti-caries protection and in addition the enamel surface can remineralize under exogenous factors. Tooth brushing is the simplest way to balance the pH and initiate remineralization processes, which allows us to emphasize that active patient involvement in respect to the dental hygiene recommendations preserve the sealant’s caries-protective effect over time [30,31,32]. The retention level of the sealing materials used in our study was above the average values attained in other studies, because they were applied by a single operator, thus diminishing the factors of inter-human variability [21,33]. In addition, in a treatment session (aided by an assistant and rendering moisture control more effective) two occlusal, antagonistic surfaces at most were approached, in order to achieve an adequate isolation of the operating field, in the conditions of collaboration with the child patient. According to the literature, in order to have preventive caries effect, dental sealant retention must be 82.5%—1 year, 70.5%—2 years, 54.3%—3 years [34]. Resin-based sealants undergo failures ranging from 5 to 10% each year because they are technique-sensitive materials; in our study group, we noticed higher values for sealant loss but accurate follow-up can control caries risk [34,35]. The variability of the results obtained by several practitioners using the same commercial product may be a reason why sealants are used with some caution [36,37]. Materials and technical means that have been used in our study can be found in a regular dental office. The average values obtained for retention allow us to consider that sealing materials applied under the normal conditions of a dental office have a good retention over time and ensure an effective protection against dental caries.

The position on dental arch and the morphological details of the first permanent molar is a factor that can influence the retention of the sealing material on the enamel surface.

When sealants were first applied in our study group, the average age of the patients was 7 years and our results showed adequate retention during the follow-up period; the null hypotheses was rejected.

At this age, the technical criteria for the application of the sealing material can be followed, in conditions of a better collaboration with the child patient [21,38]. The loss of sealing material over time is mainly due to abrasion, masticatory forces and marginal infiltration as a secondary outcome of inaccurate moisture control [26,39]. Application to erupting immature permanent teeth would be less successful, since isolation may not be provided thoroughly due to hydrophobic characteristics of resin-based materials [40,41]. During this study, it was detected that retention loss can also occur for a moisture-tolerant, resin-based and glass ionomer (GI) sealant.

Patient age alone should not be used as a major criterion for decision-making, as the caries risk on surfaces with pits and fissures might continue into adulthood and therefore, any tooth at any age could potentially benefit from sealants [21,42].

First permanent molar topography on dental arch plays an important role in dental material retention, in accordance with accurate isolation. The maxillary molars have more roots than the mandibular ones, and therefore have more surface area to dissipate loads in the trabecular bone located in this region of the mouth. The greater dissipation of occlusal forces in maxillary molars compared to mandibular molars may have also led to better sealant retention in maxillary teeth as seen in this study.

Sealant physical properties such as fluidity allow penetration in pit and fissures, but complete setting in adequate isolation must be achieved prior to ensure preventive effect [38,43]. On the other hand, mandibular molars have limited fissures and maxillary teeth are exposed to occlusal stress earlier than mandibular teeth—this might contribute to the low level of retention [32,44]. In addition to such considerations, some investigators also report that the rate of reparation is higher in mandibular teeth, since their eruption periods last longer [21,43].

## 5. Conclusions

In this study maxillary molars performed better in terms of retention when compared with mandibular molars. After 12 months, we notice that dental sealants tend to detach more frequently that after 6 or 18 months.

Resin-based sealants and glass ionomer sealants perform comparably and allow practitioners a selection in accordance with individual experience and patient-specific conditions. Sealant applied around 7 years of age perform better in terms of retention, in our study.

In the actual context of caries management, the aim of the medical model is to improve and maintain individual dental health by preventing caries from occurring with simple and effective methods adapted to child patients.

In this study, retention values after application in a usual dental office allow us to encourage the use of dental sealants with various dental materials.

## Figures and Tables

**Table 1:** Children age distribution in the study group.

Age (Average ± SD)	Median (IQR)	Range
7.11 ± 0.614 years	7 (7–7.75) years	6–8 years

**Table 2:** Dental sealant composition.

Group	Material	Producer	Composition
I	Admira seal	Voco, Cuxhaven, Germany	Ormocer dimethacrylates, inorganic microparticles, silicate fillers, fumed silica and different additives
II	Embrace Wet Bond	150320 Pulpdent Corporation, Watertown, MA, USA	Aliphatic urethane dimethacrylate, bis-methacryloyl phosphate, HEMA, trimethylolpropane trimethacrylate, water, 3% NaF, 36.6% silicon dioxide (SiO_2_)
III	Fotoseal	Remed Prodimpex SRL, Bucharest, Romania	60% dimethacrylate monomer mixture, 40% hybrid inorganic filler (colloidal silica, titanium dioxide, eutectic fluoride)
IV	Fuji GC Triage (white shade)	GC Corporation, Tokyo, Japan	Fluoroaluminium silicate glass, polyacrylic acid, polybasic carboxylic acid

**Table 3:** 1.6—Dental sealant retention assessment in relation to patient age when material was first applied.

**Status—6 Months**	**Average ± SD**	**Median (IQR)**	**Average Rank**	***p*** *
Sealant intact (*p* < 0.001 **)	7.14 ± 0.613	7 (7–8)	77.78	0.617
Lost sealant (*p* < 0.001 **)	7.33 ± 0.577	7	89.17	
**Status—12 Months**	**Average ± SD**	**Median (IQR)**	**Average Rank**	***p*** *
Sealant intact (*p* < 0.001 **)	7.14 ± 0.625	7 (7–8)	77.60	0.742
Lost sealant (*p* < 0.001 **)	7.2 ± 0.523	7 (7–7.75)	80.67	
**Status—18 Months**	**Average ± SD**	**Median (IQR)**	**Average Rank**	***p*** *
Sealant intact (*p* < 0.001 **)	7.18 ± 0.629	7 (7–8)	68.17	0.655
Lost sealant (*p* < 0.001 **)	7.11 ± 0.333	7 (7–7)	73.85	

* Mann–Whitney U Test, ** Shapiro–Wilk Test.

**Table 4:** 2.6—Dental sealant retention assessment in relation to patient age when material was first applied.

**Status—6 Months**	**Average ± SD**	**Median (IQR)**	**Average Rank**	***p*** *
Sealant intact (*p* < 0.001 **)	7.08 ± 0.589	7 (7–7)	82.00	0.015
Lost sealant (*p* = 0.001 **)	7.67 ± 0.516	8 (7–8)	123.50	
**Status—12 Months**	**Average ± SD**	**Median (IQR)**	**Average Rank**	***p*** *
Sealant intact (*p* < 0.001 **)	7.07 ± 0.612	7 (7–7)	81.93	0.274
Lost sealant (*p* < 0.001 **)	7.21 ± 0.499	7 (7–7.75)	91.21	
**Status—18 Months**	**Average ± SD**	**Median (IQR)**	**Average Rank**	***p*** *
Sealant intact (*p* < 0.001 **)	7.1 ± 0.599	7 (7–7)	78.05	0.157
Lost sealant (*p* = 0.001 **)	7.43 ± 0.535	7 (7–8)	99.36	

* Mann–Whitney U Test, ** Shapiro–Wilk Test.

**Table 5:** 3.6—Dental sealant retention assessment in relation to patient age when material was first applied.

**Status—6 Months**	**Average ± SD**	**Median (IQR)**	**Average Rank**	***p*** *
Sealant intact (*p* < 0.001 **)	7.07 ± 0.576	7 (7–7)	66.32	0.437
Lost sealant (*p* = 0.005 **)	6.94 ± 0.772	7 (6–7.75)	59.66	
**Status—12 Months**	**Average ± SD**	**Median (IQR)**	**Average Rank**	***p*** *
Sealant intact (*p* < 0.001 **)	7.04 ± 0.614	7 (7–7)	64.58	0.664
Lost sealant (*p* < 0.001 **)	7.09 ± 0.583	7 (7–7)	67.13	
**Status—18 Months**	**Average ± SD**	**Median (IQR)**	**Average Rank**	***p*** *
Sealant intact (*p* < 0.001 **)	7.13 ± 0.565	7 (7–7)	64.85	0.035
Lost sealant (*p* < 0.001 **)	6.83 ± 0.702	7 (6–7)	50.25	

* Mann–Whitney U Test, ** Shapiro–Wilk Test.

**Table 6:** 4.6—Dental sealant retention assessment in relation to patient age when material was first applied.

**Status—6 Months**	**Average ± SD**	**Median (IQR)**	**Average Rank**	***p*** *
Sealant intact (*p* < 0.001 **)	7.15 ± 0.545	7 (7–7)	63.92	0.899
Lost sealant (*p* = 0.005 **)	7.14 ± 0.900	7 (6–8)	65.43	
**Status—12 Months**	**Average ± SD**	**Median (IQR)**	**Average Rank**	***p*** *
Sealant intact (*p* < 0.001 **)	7.13 ± 0.494	7.13 ± 0.494	7.13 ± 0.494	0.373
Lost sealant (*p* < 0.001 **)	7.21 ± 0.729	7.21 ± 0.729	7.21 ± 0.729	
**Status—18 Months**	**Average ± SD**	**Median (IQR)**	**Average Rank**	***p*** *
Sealant intact (*p* < 0.001 **)	7.09 ± 0.478	7 (7–7)	58.95	0.004
Lost sealant (*p* < 0.001 **)	7.41 ± 0.747	8 (7–8)	77.70	

* Mann–Whitney U Test, ** Shapiro–Wilk Test.

## Data Availability

These data are stored on the individual files of each patient presented at the dental office, and these files are not stored in an electronic database.
